# Supplementary material for: Predicting visual function by interpreting a neuronal wiring diagram
Source: Nature. 2024 Oct 2;634(8032):113–23. doi: 10.1038/s41586-024-07953-5 (PMC11446822; doi:10.1038/s41586-024-07953-5)
Supplement: Supplementary file 6 — CRF and ERF predictions for individual TmY4 and TmY9 cells. Analogous to Supplementary Data 3, but for TmY target types. Shown are the top four monosynaptic pathways, the strongest pathway passing through each of the top ten intermediary types (ranking from Extended Data Fig. 7), and the trisynaptic pathway Tm1–TmY–Dm3–TmY (see the section entitled Prediction of spatial normalization). [file 41586_2024_7953_MOESM6_ESM.zip › DataS4/TmY9q/720575940629421386.pdf]

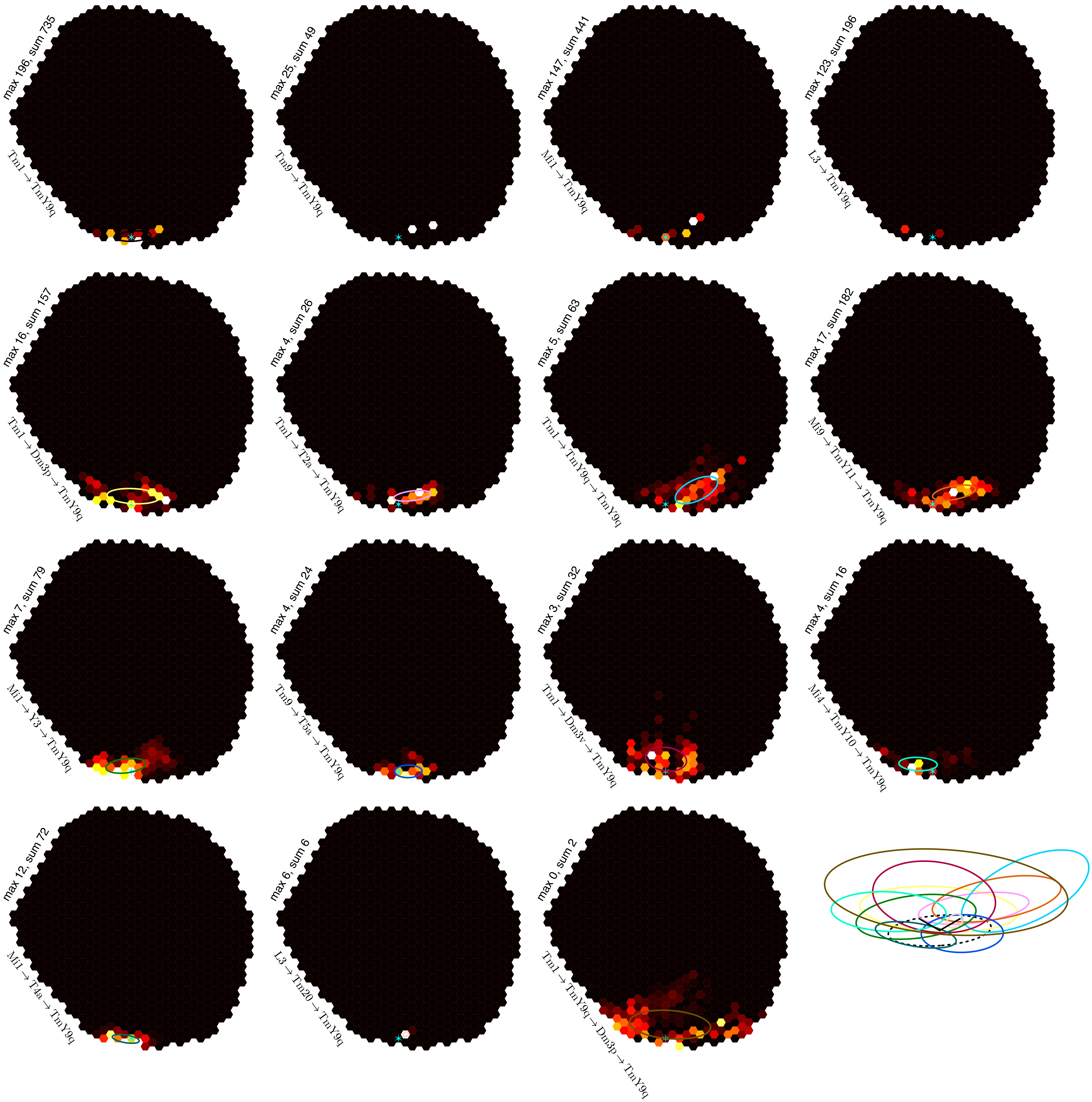

max 196, sum 735
Tm1 → TmY9q
max 25, sum 49
Tm9 → TmY9q
max 147, sum 441
Mi1 → TmY9q
max 123, sum 196
L3 → TmY9q
max 16, sum 157
Tm1 → Dm3p → TmY9q
max 4, sum 26
Tm1 → T2a → TmY9q
max 5, sum 63
Tm1 → TmY9q → TmY9q
max 17, sum 182
Mi9 → TmY11 → TmY9q
max 7, sum 79
Mi1 → Y3 → TmY9q
max 4, sum 24
Tm9 → T5a → TmY9q
max 3, sum 32
Tm1 → Dm3v → TmY9q
max 4, sum 16
Mi4 → TmY10 → TmY9q
max 12, sum 72
Mi1 → T4a → TmY9q
max 6, sum 6
L3 → Tm20 → TmY9q
max 0, sum 2
Tm1 → TmY9q → Dm3p → TmY9q
